# Update on Robotic Rectal Prolapse Treatment

**DOI:** 10.3390/jpm11080706

**Published:** 2021-07-23

**Authors:** Giampaolo Formisano, Luca Ferraro, Adelona Salaj, Simona Giuratrabocchetta, Andrea Pisani Ceretti, Enrico Opocher, Paolo Pietro Bianchi

**Affiliations:** 1 Division of General and Robotic Surgery, Dipartimento di Scienze della Salute, Università di Milano, 20142 Milano, Italy; giampaoloformisano@hotmail.com (G.F.); adelona.87@gmail.com (A.S.); simona.giuratrabocchetta@gmail.com (S.G.); PaoloPietro.Bianchi@unimi.it (P.P.B.); 2 Division of General and HPB Surgery, Dipartimento di Scienze della Salute, Università di Milano, 20142 Milano, Italy; andreapisaniceretti@yahoo.it (A.P.C.); enrico.opocher@unimi.it (E.O.)

**Keywords:** robotic surgery, robotic ventral rectopexy, rectal prolapse, pelvic organ prolapse treatment

## Abstract

Rectal prolapse is a condition that can cause significant social impairment and negatively affects quality of life. Surgery is the mainstay of treatment, with the aim of restoring the anatomy and correcting the associated functional disorders. During recent decades, laparoscopic abdominal procedures have emerged as effective tools for the treatment of rectal prolapse, with the advantages of faster recovery, lower morbidity, and shorter length of stay. Robotic surgery represents the latest evolution in the field of minimally invasive surgery, with the benefits of enhanced dexterity in deep narrow fields such as the pelvis, and may potentially overcome the technical limitations of conventional laparoscopy. Robotic surgery for the treatment of rectal prolapse is feasible and safe. It could reduce complication rates and length of hospital stay, as well as shorten the learning curve, when compared to conventional laparoscopy. Further prospectively maintained or randomized data are still required on long-term functional outcomes and recurrence rates.

## 1. Introduction

Pelvic organs prolapse, including rectal prolapse (RP), is a condition that mainly affects women in middle and advanced age and can involve both the anterior and posterior compartments. A multidisciplinary approach is traditionally required, involving urologists, gynecologists, and colorectal surgeons [1]. Depending on the anatomy and the type of prolapse, symptoms may vary from urinary or fecal incontinence to obstructed defecation, pelvic pain, and sexual dysfunction. This condition may significantly worsen the quality of life (QoL) and represent an important social and economic burden in the setting of an aging population.

Surgery is the mainstay of treating this complex disease, and several abdominal and perineal approaches have been described to date. However, since multiple options are available, treatment may be surgeon-dependent and is influenced by many factors. Therefore, a tailored, multidisciplinary approach is recommended, with abdominal procedures usually performed in younger, healthier patients and perineal procedures offered to higher-risk individuals.

External rectal prolapse or symptomatic internal rectal prolapse with rectocele or enterocele are commonly treated with ventral rectopexy in fit patients.

The abdominal approach aims to reduce rectal mobility by fixation with or without excision of the redundant colon. Rectopexy is associated with lower recurrence risk than simple rectal mobilization, with a similar rate of overall complications [2]. Fixation of the prolapsed rectum to the sacral promontory is the key to restore the physiological anatomy of the pelvic floor. This goal can be achieved by simple suturing, as first described by D. Cutait in 1959 [3], or using a mesh fixed anteriorly, posteriorly, laterally, or all over the rectum. Many techniques have been described, such as the Ripstein rectopexy, based on the anterior fixation of a mesh below the sacral promontory, or the Wells procedure, with the detachment of the lateral ligaments of the rectum.

Both these approaches are associated with a significant complication rates and are currently abandoned [4,5].

There is no evidence as to whether associated sigmoidectomy results in better functional outcomes compared to a simple rectopexy. Resection rectopexy is thought to improve complaints of constipation, reducing the possible kinking of the redundant colon. However, it is a matter of fact that the creation of an anastomosis may increase the risk of severe complications [6,7,8]. Ventral rectopexy is typically performed laparoscopically and involves the anterior placement of a mesh to the sacral promontory, as described by D’Hoore [9]. It is favored over posterior mesh rectopexy since it reduces autonomic nerve injuries by avoiding postero-lateral dissection of the rectum. This approach thus reduces impairment of rectal motility that could potentially and ultimately lead to ongoing functional disfunction and impaired quality of life [10,11].

Since the introduction of the minimally invasive treatment for rectal prolapse in the early 90 s [12], the uptake of laparoscopy has been progressively growing to treat this condition. The benefits of the minimally invasive approach are well known in terms of faster recovery and normal return to daily activities, lower morbidity, decreased postoperative pain, shorter length of stay, and lower blood loss and the laparoscopic approach as the preferred technique has been recommended by several authors [13,14,15,16]. Laparoscopy has shown similar outcomes compared to the open technique for the surgical treatment of rectal prolapse [14,17]. A meta-analysis by Sajid et al. in 2010 reported no statistically significant difference between 688 patients treated with an open or laparoscopic approach in terms of recurrence, functional outcomes, and complication rate. Moreover, they reported a shorter length of hospital stay in the laparoscopic group [18]. However, the laparoscopic approach can be challenging, especially in the deep and narrow pelvis or in the setting of morbid obesity.

Since its introduction, the uptake of robotic surgery in several fields of general surgery has constantly grown. Robotic assistance is rapidly increasing in pelvic floor surgery because of its advantages in complex maneuvers such as dissection and intracorporeal suturing in the deep narrow pelvis. The technical features of the available robotic platforms may potentially overcome the limitations of conventional laparoscopy, thanks to enhanced dexterity, a stable optical platform, and exposure (third arm) that allows for a “precision” surgery to be performed. Adequate traction and counter traction allow for optimal surgical field exposure following embryological planes with minimal tissue trauma and blood loss [19]. Moreover, it has the potential of shortening the learning curve even regarding rectal mesh rectopexy, as demonstrated in other surgical procedures [20,21]. This study aims to describe the surgical technique of robotic ventral rectopexy and to review the available literature on intraoperative, short-term postoperative, and long-term functional outcomes.

## 2. Surgical Technique

The patient is placed in the lithotomy position. The arms are folded at the sides, taking care to provide adequate padding along the pressure points. An anti-slipping soft foam dedicated pad should be placed directly under the patient to conduct the operation safely. This device facilitates the steep Trendelenburg position often required to ensure adequate pelvic exposure.

**Figure 1:**
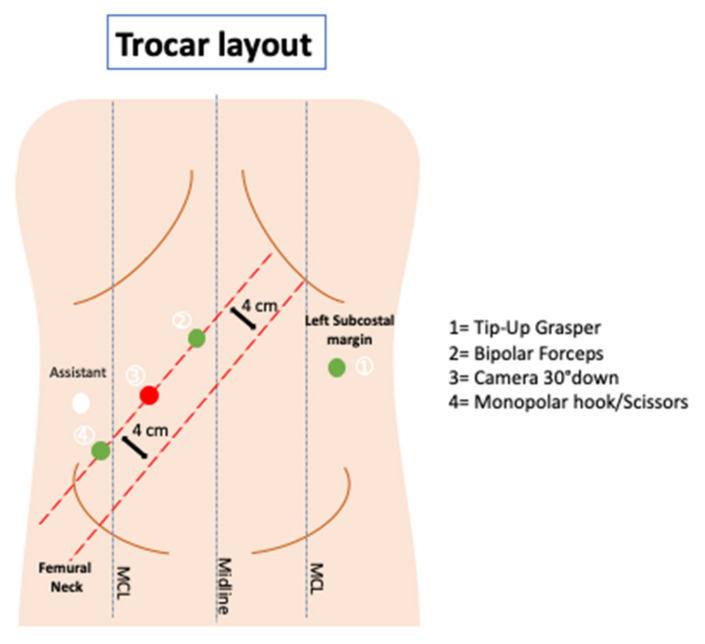
Trocar layout.

A Verres needle is inserted at Palmer’s point in the left hypochondrium to create the pneumoperitoneum. Access to the peritoneal cavity is achieved by a first assistant 12-mm optical trocar placed in the right flank under direct vision. The costo-femoral line is the landmark used to place three 8 mm robotic trocars along a parallel straight line, approximately 4 cm lateral to the previous one. Finally, an additional 8 mm robotic trocar is positioned in the left flank. Figure 1 shows the trocar layout. Limited laparoscopic lysis is performed when adhesions are encountered to permit the safe positioning of the robotic trocars; the adhesiolysis is then completed under robotic assistance.

**Figure 2:**
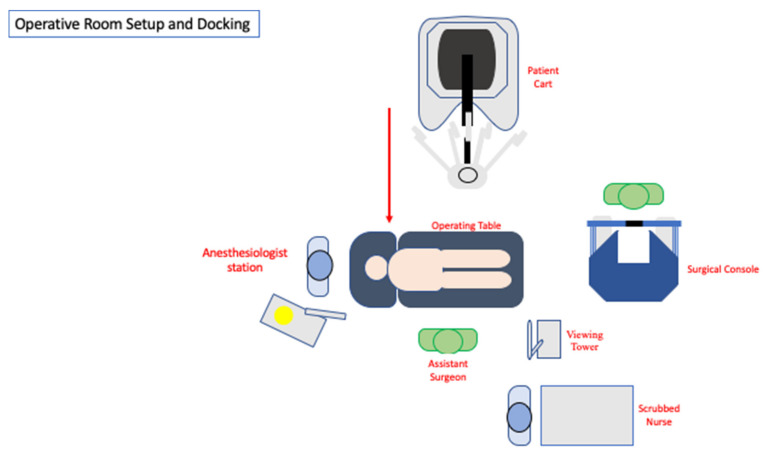
Operative room setup.

The patient is then positioned in a steep Trendelenburg and right tilt (20–25°), allowing the small bowel to be displaced under gravity, thus obtaining a good surgical field exposure. Next, the Da Vinci Xi^®^ surgical system (Intuitive Surgical, Sunnyvale, CA, USA) is docked from the patient’s left side. A full-robotic procedure is performed, with the assistant surgeon and scrub nurse standing on the patient’s right side (Figure 2).

Tip-up grasper, bipolar forceps, and monopolar cautery hook/scissors (according to operating surgeon’s preference) are mounted on robotic arm 1 (R1), arm 2 (R2), and arm 4 (R4), respectively. Robotic arm 3 (R3) is used for the 30° down scope.

The sigmoid colon is grasped and elevated anteriorly and cranially by the tip-up grasper in R1.

The peritoneum is entered by sharp dissection starting at the base of the rectosigmoid mesentery, identifying the avascular areolar plane along the sacral promontory. The right hypogastric nerve plexus and the ureter are then identified and preserved. The rectovaginal septum represents the limit to conduct the peritoneal incision.

At the level of the pouch of Douglas, the peritoneal incision is continued from right to left over the ventral aspect of the rectum.

A Breisky uterine and vaginal manipulator can identify and lift the posterior vaginal wall, thus facilitating the dissection along the anterior rectal wall. At this stage, the third arm is used as a retractor deep in the pelvis (lifting the posterior vaginal wall, once identified), and the assistant’s atraumatic grasper lifts the rectum. The rectovaginal septum is entered, and anterior dissection is carried out all the way down to the levator ani plane, as inferiorly as possible, and laterally to the cardinal ligaments and pelvic sidewalls. The rectum is fully mobilized anteriorly, while the posterior and lateral attachments are left intact to preserve the autonomic nerves and optimize functional outcomes in the postoperative period.

**Figure 3:**
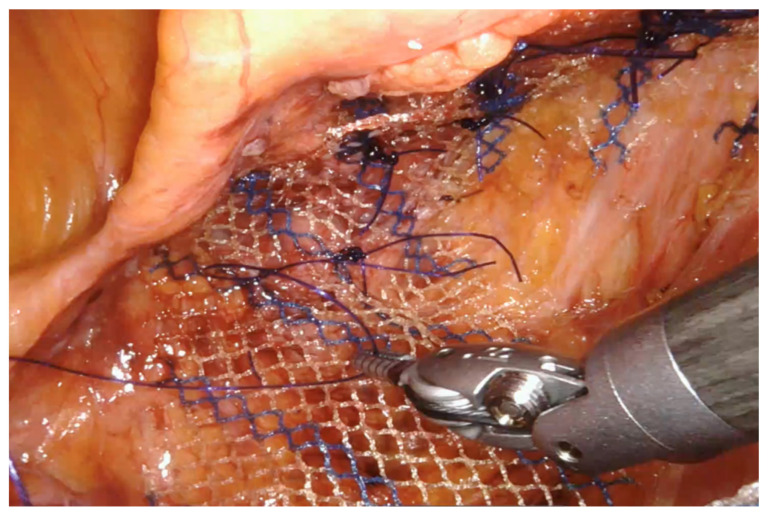
The mesh (macroporous polipropilene) is secured distally at the level of the anterior rectal wall with permanent sutures.

**Figure 4:**
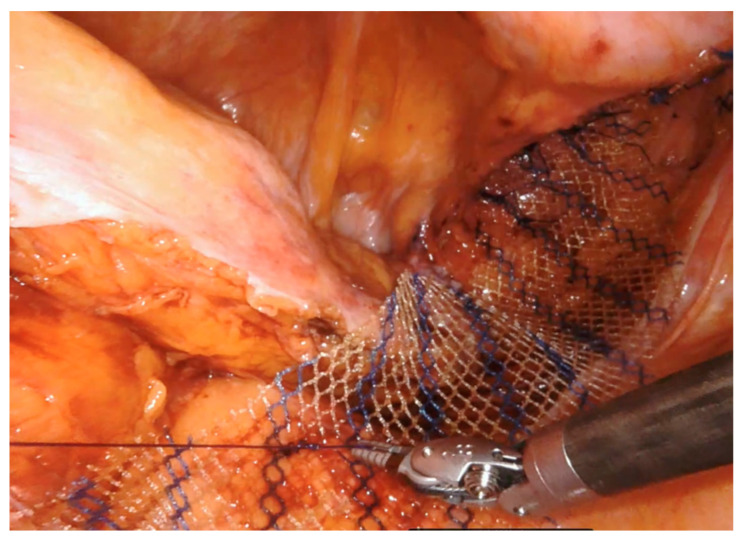
The mesh (macroporous polipropilene) is secured cranially at the level of the sacral promontory with permanent sutures.

**Figure 5:**
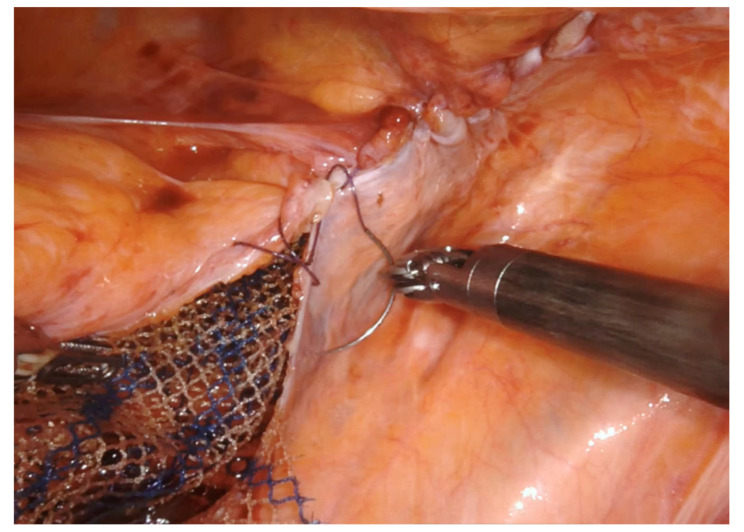
The peritoneum is closed with absorbable barbed running suture.

A 14–18 cm long, 3–4 cm wide, light-weight macroporous polypropylene mesh is inserted into the abdominal cavity through the 12 mm assistant port. Biologic and titanium-coated polypropylene mesh can also be used. The mesh is positioned along the anterior wall of the rectum caudally and at the level of the sacral promontory cranially (Figure 3 and Figure 4). Four interrupted stitches are used to secure the mesh along the anterior distal extraperitoneal surface of the rectum, using a 2-0 non-absorbable monofilament. The mesh is then fixated at the level of the sacral promontory with a 2-0 non-absorbable monofilament interrupted suture, taking care to preserve both the presacral venous plexus and the hypogastric nerves. The peritoneum is then closed with a reabsorbable barbed running suture (Figure 5). No drain is routinely left in place. Trocars are removed under direct vision, and the fascial defect of the 12-mm assistant port is closed with absorbable sutures.

## 3. Discussion and Literature Review

Currently, minimally invasive surgery has widespread indications in colorectal surgery, with the robotic-assisted platform gaining extensive consensus due to its technical advantage in narrow and limited spaces [22].

Among all the various surgical options available for rectal prolapse treatment, ventral mesh rectopexy is the only technique that does not require a full rectal mobilization, with a limited anterior rectal preparation. This procedure has become the standard of care for patients with full-thickness rectal prolapse and deep enterocele [22,23,24]. However, it requires a good dissection of the anterior rectal surface from the prostate or the vagina and the fixation of a mesh within the narrow confines of the pelvis.

An important objective of rectal prolapse surgical treatment is to resolve or improve the functional symptoms (e.g., fecal incontinence, constipation, pain) by correcting the underlying anatomical defect. This goal should be obtained with an acceptable recurrence rate and at a reasonable cost.

The laparoscopic ventral rectopexy (LVR) is the treatment of choice for rectal prolapse nowadays [24]. LVR’s use reflects widespread laparoscopy diffusion, although surgical robots have gained broad availability and have more indications in the modern surgical scenario.

To date, few studies have reported the outcomes of robotic ventral rectopexy (RVR), with most consisting of a small sample size. However, data in the literature reports that the robotic approach to rectal prolapse is feasible and safe, with outcomes almost on a par with the laparoscopic and open techniques [19,25,26].

In this section, we report on the currently available data on RVR, analyze the short-term, functional outcomes and recurrence of this approach, and look at data comparing the robotic approach with the laparoscopic approach.

### 3.1. Intraoperative and Short-Term Post-Operative Outcomes

Most authors report on the feasibility and safety of RVR, mainly due to the capability of the robot to conduct a fine dissection in deep and narrow space [27,28,29].

Features such as three-dimensional vision, restorable eye-hand-targeting, absence of depth misperception, tremor elimination, better definition of surgical planes, and robotic instrumentation wristing may facilitate the surgeon performing a correct anatomical dissection and mesh fixation in the pelvis [19].

Ventral mesh rectopexy is ideally suited for robotic surgery. The robotic platform ameliorates the visualization of the pelvis, facilitating the dissection and the suturing capability in narrow and confined spaces, allowing an optimal mesh placement to the rectovaginal septum. Indeed, the fixation of the mesh to the pelvic structures is technically more accessible, thus fastening the learning curve, with approximately twenty cases described to gain proficiency with the robotic technique compared to almost one hundred cases of the laparoscopic approach [30,31].

A recent systematic review by Albayati et al. [22] of five prospective cohort studies and one randomized controlled trial reports a significant increase in operating time for RVR compared to the laparoscopic approach. This finding is similar to that of a previous meta-analysis conducted by Ramage et al. [28]. A longer operative time is one of the main criticisms and on-vogue topics by the detractors of robotic surgery. However, it must be taken into account that these series usually show the outcomes of experienced laparoscopic surgeons compared to those of surgeons at the beginning of their robotic experience [25,30,32,33]. Indeed, recent series have described that the mean operative time for robotic rectopexy decreases with increased caseloads and experience [34,35]. These data have been confirmed by a recent metanalysis showing a non-significant trend towards longer operative times of robotic vs. laparoscopic ventral mesh rectopexy [36].

The previous systematic reviews report no statistically significant reduction of conversion rate associated with RVR [22,28]. This may be the consequence either of the exiguity of the pooled data or may be explained by the different operating surgeons’ experience. Previous reviews and metanalysis of rectal prolapse treatment also describe an unclear benefit of reducing the conversion rate [36,37,38]. However, data in the literature show promising results in lowering the conversion rate of the robotic approach compared to open surgery in colorectal surgery [39,40,41].

Data in the literature show that RVR is safe and effective. Munz et al. [42], in the early 2000s, described no major complications in six patients treated robotically for rectal prolapse.

Germain et al. [35], in 2014, reported a morbidity rate of 1.7% after seventy-seven RVR. They did not reach statistical significance in the complication rate between elderly and young patients [35]. Recently, van Iersel et al. [43], in a large meta-analysis of twenty-seven studies, outlines post-operative morbidity ranging from 4.5% to 11% for the RVR reports, compared to the 0% to 23.5 that occurred in the LVR series. Bao et al. have recently documented a significant decrease in post-operative complications by 0.45 (95% confidence interval (CI), 0.24–0.83, *p* = 0.009) in the RVR group compared to the LVR group, with eight studies included in their metanalysis [38].

Robotic surgery is often criticized regarding the lack of tactile feedback during the manipulation of the anatomical structures, resulting in uncontrolled tractions leading to possible organ injuries during the procedure. In our long-lasting colorectal robotic experience, the misperception of force feedback during the dissection of the rectum from the sacral promontory and beyond is overcome thanks to increased visual feedback, which helps to recognize the anatomical landmarks better, facilitating the dissection and the respect of the hypogastric nerves, presacral venous plexus, and ureters. Moreover, the fixed third arm used for retraction permits a stable exposition of the surgical field, allowing a fine dissection throughout the operation. These features optimize the dissection along the embryological planes, as occur during total mesorectal excision.

Robotic surgery is associated with higher hospital costs compared to the laparoscopic technique. Several studies have shown how robotic surgery is related to higher costs than laparoscopy in rectal prolapse surgical treatment [44,45].

However, a recent study shows how RVR’s expenditure is almost comparable to that of the laparoscopic approach after adjusting the costs for improved health-related quality of life [46]. Moreover, in their recent series, Albayati et al. [22] show a shorter length of hospital stay after RVR, which is a common finding after robotic surgery, thus increasing the cost-effectiveness and decreasing the overall expenditure of robotic surgery procedures [47]. The shorter length of stay could offset higher equipment expenditure and theatre costs related to robotic surgery, with faster recovery probably related to reduced pain, bleeding, and complications due to a more precise pelvic dissection [36].

### 3.2. Functional Outcomes

Ventral mesh rectopexy is associated with lower constipation and fecal incontinence than other trans-abdominal or perineal procedures [48,49].

This technique was initially ideated to reduce post-operative constipation related to the posterolateral detachment of the rectum, thus minimizing autonomic nerve injuries [9].

A limited anterior rectal dissection is associated with a minimal risk of damaging the parasympathetic fibers of the hypogastric plexus, with a reduced rate of post-operative functional impairments, as demonstrated in several studies [50].

De Hoog et al. [51] report a median Cleveland clinic constipation score (CCCS) gain of 3.2 points after RVR. This series reports no statistical difference in the functional outcomes (CCCS, Wexner Incontinence Score, Impact on daily life-score IDL) between the open, laparoscopic, and robotic approaches. Similar results are reported by other studies [19,42,44].

A recent clinical trial by Mehmood et al. [26] shows how the post-operative Wexner incontinence score is significantly lower in the RVR group compared to the laparoscopic group. Furthermore, they report that the Short Form Health Survey 36 (SF-36) questionnaires reach higher scoring with the robotic approach compared to laparoscopy [26]. Additionally, Mantoo et al. [33] report a significant improvement for obstructed defecation after RVR. These data, however, were not confirmed by a recent metanalysis that showed lower mean Wexner and fecal incontinence scores in the RVR group but without reaching statistical significance [38].

In fact, the small size of pooled data and the short duration of follow-up reported in those studies make it difficult to derive any conclusion from the available literature. Moreover, patients’ heterogeneity, different standards of outcome detection, and the lack of a systematic approach adopted for most studies need to be considered when analyzing these results.

### 3.3. Recurrences

Ventral mesh rectopexy shows similar recurrence rates and less functional post-operative complications than other abdominal approaches for rectal prolapse [48,49,52]. According to current data, the recurrence rate following RVR ranges between 0% up to 20% compared to 0% to 26.7% with the laparoscopic approach, never reaching statistical significance [22,43]. The use of a mesh to lift the middle compartment of the pelvis has been subject to discussion in recent years [53]. However, recent studies report a low rate of mesh-related complications, with mesh erosion percentage raging up to 4% of complications following ventral mesh rectopexy [54,55]. There are many different types of mesh available to use, generally divided into synthetic and biological. Synthetics are usually lightweight or heavyweight polypropylene mesh, with polyester and expanded polytetrafluoroethylene not being recommended due to a high rate of post-operative recurrence [24,31].

Biological meshes have been developed to reduce the risk of mesh erosion and infection thanks to the time-related deterioration with the regeneration of host tissue. Conversely, the degradation of the material may be associated with a higher percentage of recurrence. However, current data do not show a significant difference in both mesh-related complications and recurrence rate between the synthetic and biological grafts, suggesting the use of the latter in high-risk patients (diabetics, smokers, with previous pelvic radiation, with inflammatory bowel disease, with intraoperative finding of rectum or vaginal leak) [43,55,56].

Again, no consistent and robust long-term data are available to draw firm conclusions.

## 4. Conclusions

Robotic surgery is a safe and feasible approach for the treatment of rectal prolapse that may potentially lower complication rates and length of hospital stay, as well as shorten the learning curve thanks to its technological features. Consistent long-term prospective or randomized data are needed on recurrence and functional improvement of robotic surgical treatment of rectal prolapse compared to laparoscopic rectopexy.

## Data Availability

Not applicable.

## References

[1] Wijffels N.A., Collinson R., Cunningham C., Lindsey I. (2009). What is the natural history of internal rectal prolapse?. Color. Dis..

[2] Karas J.R., Uranues S., Altomare D.F., Sokmen S., Krivokapic Z., Hoch J., Bartha I., Bergamaschi R. (2011). No Rectopexy Versus Rectopexy Following Rectal Mobilization for Full-Thickness Rectal Prolapse: A Randomized Controlled Trial. Dis. Colon Rectum.

[3] Cutait D. (1959). Sacro-promontory fixation of the rectum for complete rectal prolapse. Proc. R Soc. Med..

[4] Hrabe J., Gurland B. (2016). Optimizing Treatment for Rectal Prolapse. Clin. Colon Rectal Surg..

[5] Novell J.R., Osborne M.J., Winslet M.C., Lewis A.A. (1994). Prospective randomized trial of Ivalon sponge versus sutured rectopexy for full-thickness rectal prolapse. Br. J. Surg..

[6] Senapati A., Gray R., Middleton L.J., Harding J., Hills R., Armitage N.C.M., Buckley L., Northover J.M.A. (2013). The PROSPER Collaborative Group PROSPER: A randomised comparison of surgical treatments for rectal prolapse. Color. Dis..

[7] Luukkonen P., Mikkonen U. (1992). Abdominal rectopexy with sigmoidectomy vs. rectopexy alone for rectal prolapse: A prospective, randomized study. Int. J. Color. Dis..

[8] McKee R., Lauder J., Poon F. (1992). A prospective randomized study of abdominal rectopexy with and without sig-moidectomy in rectal prolapse. Surg. Gynecol. Obs..

[9] D’Hoore A., Cadoni R., Penninckx F. (2004). Long-term outcome of laparoscopic ventral rectopexy for total rectal prolapse. BJS.

[10] Varma J.S. (1992). Autonomic influences on colorectal motility and pelvic surgery. World J. Surg..

[11] El Muhtaseb M.S., Bartolo D.C., Zayiae D., Salem T. (2014). Colonic transit before and after resection rectopexy for full-thickness rectal prolapse. Tech. Coloproctol..

[12] Kim D.-S., Tsang C.B.S., Wong D.W., Lowry A.C., Goldberg S.M., Madoff R.D. (1999). Complete rectal prolapse. Dis. Colon Rectum.

[13] Kairaluoma M.V., Viljakka M.T., Kellokumpu I.H. (2003). Open vs. Laparoscopic Surgery for Rectal Prolapse. Dis. Colon Rectum.

[14] Kariv Y., Delaney C.P., Casillas S., Hammel J., Nocero J., Bast J., Brady K., Fazio V.W., Senagore A.J. (2005). Long-term outcome after laparoscopic and open surgery for rectal prolapse. Surg. Endosc..

[15] Purkayastha S., Tekkis P., Athanasiou T., Aziz O., Paraskevas P., Ziprin P., Darzi A. (2005). A Comparison of Open vs. Laparoscopic Abdominal Rectopexy for Full-Thickness Rectal Prolapse: A Meta-Analysis. Dis. Colon Rectum.

[16] Kessler H., Hohenberger W. (2005). Laparoscopic Resection Rectopexy for Rectal Prolapse. Dis. Colon Rectum.

[17] Solomon M.J., Young C.J., Eyers A., Roberts R.A. (2002). Randomized clinical trial of laparoscopic versus open abdominal rectopexy for rectal prolapse. BJS.

[18] Sajid M.S. (2010). Open versus laparoscopic repair of fullthickness rectal prolapse: A re-meta-analysis. Colorectal Dis..

[19] Ayav A., Bresler L., Hubert J., Brunaud L., Boissel P. (2005). Robotic-assisted pelvic organ prolapse surgery. Surg. Endosc..

[20] Trastulli S., Cirocchi R., Desiderio J., Coratti A., Guarino S., Renzi C., Corsi A., Boselli C., Santoro A., Minelli L. (2015). Robotic versus Laparoscopic Approach in Colonic Resections for Cancer and Benign Diseases: Systematic Review and Meta-Analysis. PLoS ONE.

[21] Formisano G., Esposito S., Coratti F., Giuliani G., Salaj A., Bianchi P.P. (2019). Structured training program in colorectal surgery: The robotic surgeon as a new paradigm. Minerva Chir..

[22] Albayati S., Chen P., Morgan M.J., Toh J.W.T. (2019). Robotic vs. laparoscopic ventral mesh rectopexy for external rectal prolapse and rectal intussusception: A systematic review. Tech. Coloproctol..

[23] Faucheron J.-L., Trilling B., Girard E., Sage P.-Y., Barbois S., Reche F. (2015). Anterior rectopexy for full-thickness rectal prolapse: Technical and functional results. World J. Gastroenterol..

[24] Van Der Schans E.M., Paulides T.J.C., Wijffels N.A., Consten E.C.J. (2018). Management of patients with rectal prolapse: The 2017 Dutch guidelines. Tech. Coloproctol..

[25] Ruurda J.P., Visser P.L., Broeders I.A. (2003). Analysis of procedure time in robot-assisted surgery: Comparative study in laparo-scopic cholecystectomy. Comput. Aided. Surg..

[26] Mehmood R.K., Parker J., Bhuvimanian L., Qasem E., Mohammed A.A., Zeeshan M., Grugel K., Carter P., Ahmed S. (2014). Short-term outcome of laparoscopic versus robotic ventral mesh rectopexy for full-thickness rectal prolapse. Is robotic superior?. Int. J. Color. Dis..

[27] Gurland B. (2014). Ventral Mesh Rectopexy. Dis. Colon Rectum.

[28] Ramage L., Georgiou P., Tekkis P., Tan E. (2015). Is robotic ventral mesh rectopexy better than laparoscopy in the treatment of rectal prolapse and obstructed defecation? A meta-analysis. Tech. Coloproctol..

[29] Faucheron J.-L., Trilling B., Barbois S., Sage P.-Y., Waroquet P.-A., Reche F. (2016). Day case robotic ventral rectopexy compared with day case laparoscopic ventral rectopexy: A prospective study. Tech. Coloproctol..

[30] Mäkelä-Kaikkonen J., Rautio T., Pääkkö E., Biancari F., Ohtonen P., Mäkelä J. (2016). Robot-assisted versus laparoscopic ventral rectopexy for external, internal rectal prolapse and enterocele: A randomised controlled trial. Color. Dis..

[31] MacKenzie H., Dixon A.R. (2014). Proficiency gain curve and predictors of outcome for laparoscopic ventral mesh rectopexy. Surgery.

[32] Mäkelä-Kaikkonen J., Rautio T., Klintrup K., Takala H., Vierimaa M., Ohtonen P., Mäkelä J. (2013). Robotic-assisted and laparoscopic ventral rectopexy in the treatment of rectal prolapse: A matched-pairs study of operative details and complications. Tech. Coloproctol..

[33] Mantoo S., Podevin J., Regenet N., Rigaud J., Lehur P.-A., Meurette G. (2013). Is robotic-assisted ventral mesh rectopexy superior to laparoscopic ventral mesh rectopexy in the management of obstructed defaecation?. Color. Dis..

[34] Moghadamyeghaneh Z., Hanna M.H., Hwang G., Carmichael J.C., Mills S.D., Pigazzi A., Stamos M.J. (2015). Surgical management of rectal prolapse: The role of robotic surgery. World J. Surg. Proced..

[35] Germain A., Perrenot C., Scherrer M.-L., Ayav C., Brunaud L., Ayav A., Bresler L. (2014). Long-term outcome of robotic-assisted laparoscopic rectopexy for full-thickness rectal prolapse in elderly patients. Color. Dis..

[36] Flynn J., Larach J.T., Kong J.C.H., Warrier S.K., Heriot A. (2021). Robotic versus laparoscopic ventral mesh rectopexy: A systematic review and meta-analysis. Int. J. Color. Dis..

[37] Rondelli F., Bugiantella W., Villa F., Sanguinetti A., Boni M., Mariani E., Avenia N. (2014). Robot-assisted or conventional laparoscoic rectopexy for rectal prolapse? Systematic review and meta-analysis. Int. J. Surg..

[38] Bao X., Wang H., Song W., Chen Y., Luo Y. (2021). Meta-analysis on current status, efficacy, and safety of laparoscopic and robotic ventral mesh rectopexy for rectal prolapse treatment: Can robotic surgery become the gold standard?. Int. J. Color. Dis..

[39] Prete F., Pezzolla A., Prete F., Testini M., Marzaioli R., Patriti A., Jimenez-Rodriguez R.M., Gurrado A., Strippoli G.F.M. (2018). Robotic Versus Laparoscopic Minimally Invasive Surgery for Rectal Cancer. Ann. Surg..

[40] Bhama A.R., Obias V., Welch K.B., Vandewarker J.F., Cleary R.K. (2015). A comparison of laparoscopic and robotic colorectal surgery outcomes using the American College of Surgeons National Surgical Quality Improvement Program (ACS NSQIP) database. Surg. Endosc..

[41] Formisano G., Giuliani G., Salaj A., Salvischiani L., Ferraro L., De Luca M., Bianchi P.P. (2021). Robotic elective colectomy for diverticular disease: Short-term outcomes of 80 patients. Int. J. Med Robot. Comput. Assist. Surg..

[42] Munz Y., Moorthy K., Kudchadkar R., Hernandez J., Martin S., Darzi A., Rockall T. (2004). Robotic assisted rectopexy. Am. J. Surg..

[43] Van Iersel J.J., Paulides T.J.C., Verheijen P.M., Lumley J.W., Broeders I., Consten E.C.J. (2016). Current status of laparoscopic and robotic ventral mesh rectopexy for external and internal rectal prolapse. World J. Gastroenterol..

[44] Heemskerk J., De Hoog D.E., Van Gemert W.G., Baeten C.G., Greve J.W., Bouvy N.D. (2007). Robot-assisted vs. conventional laparo-scopic rectopexy for rectal prolapse: A comparative study on costs and time. Dis. Colon Rectum..

[45] Perrenot C., Germain A., Scherrer M.-L., Ayav A., Brunaud L., Bresler L. (2013). Long-term Outcomes of Robot-assisted Laparoscopic Rectopexy for Rectal Prolapse. Dis. Colon Rectum..

[46] Mäkelä-Kaikkonen J., Rautio T., Ohinmaa A., Koivurova S., Ohtonen P., Sintonen H., Mäkelä J. (2019). Cost-analysis and quality of life after laparoscopic and robotic ventral mesh rectopexy for posterior compartment prolapse: A randomized trial. Tech. Coloproctol..

[47] Salman M., Bell T., Martin J., Bhuva K., Grim R., Ahuja V. (2013). Use, cost, complications, and mortality of robotic versus non-robotic general surgery procedures based on a nationwide database. Am. Surg..

[48] Madiba T.E., Baig M.K., Wexner S.D. (2005). Surgical Management of Rectal Prolapse. Arch. Surg..

[49] Cadeddu F., Sileri P., Grande M., De Luca E., Franceschilli L., Milito G. (2011). Focus on abdominal rectopexy for full-thickness rectal prolapse: Meta-analysis of literature. Tech. Coloproctol..

[50] Speakman C.T.M., Madden M.V., Nicholls R.J., Kamm M.A. (2005). Lateral ligament division during rectopexy causes constipation but prevents recurrence: Results of a prospective randomized study. BJS.

[51] De Hoog D.E., Heemskerk J., Nieman F.H., Van Gemert W.G., Baeten C.G., Bouvy N.D. (2009). Recurrence and functional results after open versus conventional laparoscopic versus robot-assisted laparoscopic rectopexy for rectal prolapse: A case-control study. Int. J. Colorectal Dis..

[52] Gosselink M.P., Adusumilli S., Gorissen K.J., Fourie S., Tuynman J.B., Jones O.M., Cunningham C., Lindsey I. (2013). Laparoscopic ventralrectopexy for fecal incontinence associated with high-grade internal rectal prolapse. Dis. Colon Rectum..

[53] Food and Drug Administration (2011). FDA Safety Communication: Urogynecologic Surgical Mesh: Update on the Safety and Effectiveness of Transvaginal Placement for Pelvic Organ Prolapse. Rev. Lit. Arts Am..

[54] Smart N., Pathak S., Boorman P., Daniels I.R. (2013). Synthetic or biological mesh use in laparoscopic ventral mesh rectopexy—A systematic review. Color. Dis..

[55] Evans C., Stevenson A.R.L., Sileri P., Mercer-Jones M.A., Dixon A.R., Cunningham C., Jones O.M., Lindsey I. (2015). A Multicenter Collaboration to Assess the Safety of Laparoscopic Ventral Rectopexy. Dis. Colon Rectum.

[56] Mercer-Jones M.A., D’Hoore A., Dixon A.R., Lehur P., Lindsey I., Mellgren A., Stevenson A.R.L. (2014). Consensus on ventral rectopexy: Report of a panel of experts. Color. Dis..

